# Pituitary metastasis of lung adenocarcinoma: Case report and literature review

**DOI:** 10.1016/j.ijscr.2020.01.013

**Published:** 2020-01-23

**Authors:** Aminah Alhashem, Mahmoud Taha, Ali Almomen

**Affiliations:** a Imam Abdulrahman Bin Faisal University, Saudi Arabia; b Department of Neurosurgery, King Fahad Specialist Hospital, Dammam, Saudi Arabia; c Department of Otorhinolaryngology, King Fahad Specialist Hospital, Dammam, Saudi Arabia

**Keywords:** Pituitary metastasis, Sellar mass, Adenocarcinoma, Endoscopic trans-sphenoidal surgery, Case report

## Abstract

**Introduction:**

Pituitary metastasis is rare, and it is the least common site of intracranial metastases. It is mostly asymptomatic but can present with diabetes insipidus, headache, ophthalmoplegia, visual disturbance and anterior pituitary dysfunction and in majority of cases patients known to have a primary malignancy.

**Case Presentation:**

This study presents a 54-year-old male presented with a few months history of headache and drooping of both eyelids and after proper investigations and endoscopic resection of the sellar mass the diagnosis was confirmed to be pituitary metastasis of lung adenocarcinoma.

**Conclusion:**

Diagnosis and management of pituitary metastases are complex and depend on many factors. Endoscopic surgical resection and debulking alleviates symptoms and provides the definitive diagnosis which has great importance in cases with clinical presentation that does not strongly point to pituitary metastasis to avoid unnecessary radiotherapy and chemotherapy when possible.

## Highlights

• Pituitary metastasis is rare, and it is the least common intracranial site of metastases.
• Majority of cases of pituitary metastasis the patient is known to have a primary malignancy.
• The clinical presentation and radiological features of pituitary metastasis are not specific making diagnosis difficult.
• Management may include endoscopic trans-sphenoidal resection, chemotherapy, radiotherapy and hormone replacement.
• Definitive diagnosis can be achieved through surgical resection.

## 1 Introduction

Pituitary masses are one of the most common intracranial lesions which can be found in about 20 % of the population as an incidental finding, however, most of them are benign pituitary adenomas [1]. Metastasis to the pituitary gland is considered rare. Breast and lung cancer are the most common primary site and even though they are known to metastasize to the brain pituitary gland is the least common intracranial site [2,3]. Pituitary metastases are mostly asymptomatic, and most patients die prior to its diagnosis, however, the most common presenting symptoms are diabetes insipidus, headache, ophthalmoplegia, visual disturbance or anterior pituitary dysfunction [[2], [3], [4]]. It is difficult to distinguish pituitary metastasis from other pituitary lesions based on clinical and radiological features due to the lack of specific radiological features [2]. The diagnosis usually relies on the history of known primary malignancy. Management is complex and it depends on many factors and may include surgery, radiotherapy and chemotherapy [2,5].

This case report has been reported in line with the SCARE criteria [6].

## 2 Case presentation

**Fig. 1:**
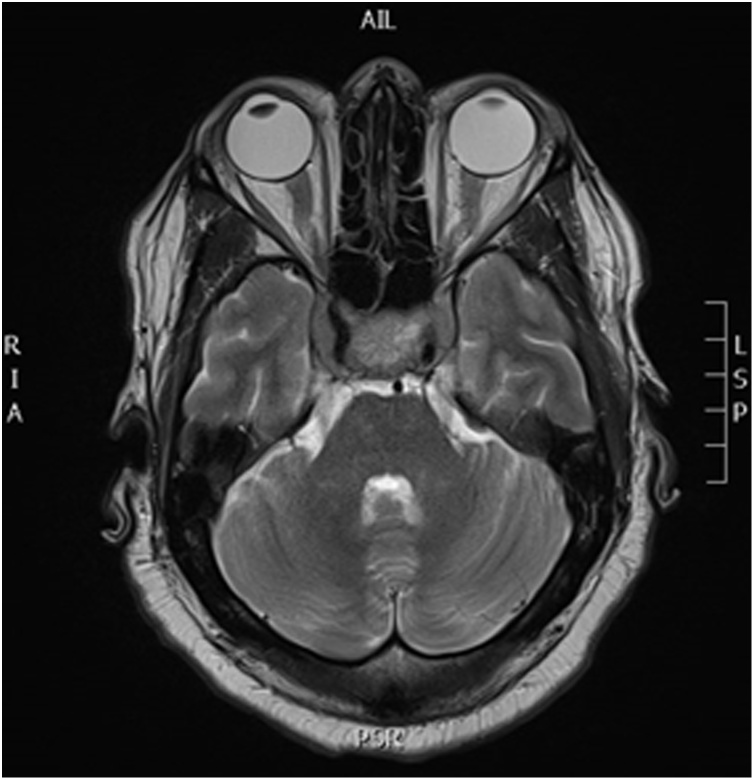
MRI brain T2 axial view showing an isotense sellar and suprasellar lesion.

**Fig. 2:**
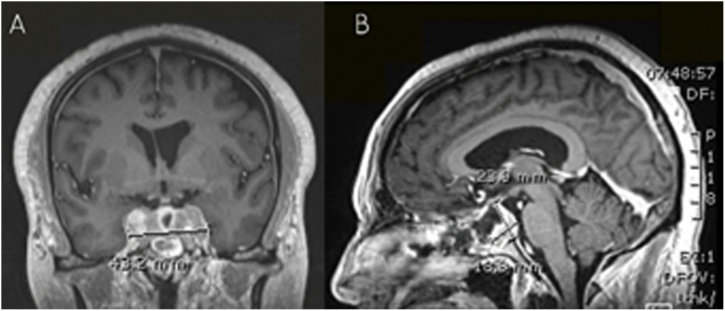
MRI brain T1 A) coronal view B) sagittal view showing sellar and suprasellar enhancing lesion replacing the pituitary gland with extension along the pituitary stalk and invasion of the cavernous sinuses measuring 4.3 × 2.3 × 1.8 cm.

**Fig. 3:**
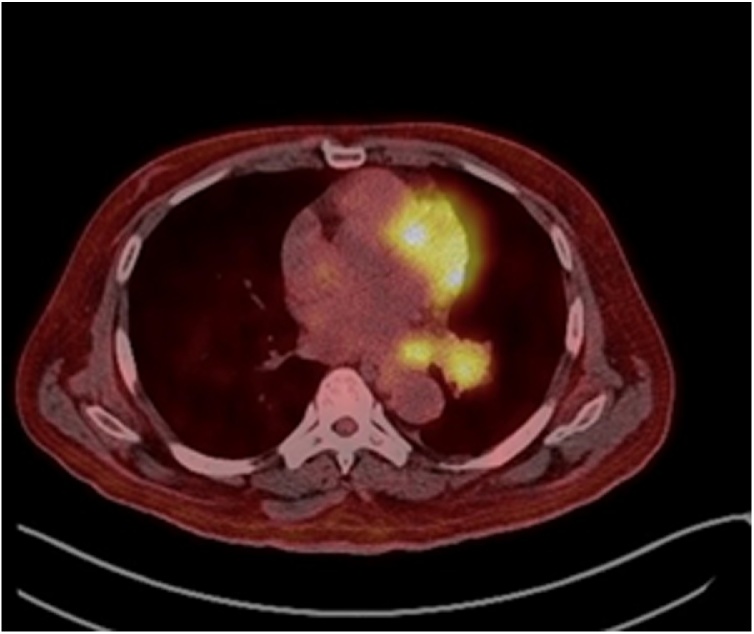
FDG PET scan axial view showing left parahilar mass in the left lower lung lobe most likely lung cancer.

A 54-year-old male known to have type II diabetes mellitus, hypertension, dyslipidemia and chronic kidney disease stage III. He presented complaining of on and off episodes of headache mainly at the frontal area for three months duration, which was accompanied with gradually progressing drooping of his left eyelid initially and then involving the right eyelid as well. It was not associated with visual impairment, seizures, fever, weakness or change in sensation and there is no history of trauma. His symptoms were not proceeded by an upper respiratory tract infection or a febrile illness. He also reported an occasional dry cough and he noticed some weight loss; however, it was not measured. The cough was not associated with chest pain, shortness of breath or hoarseness of voice. Upon examination, the patient was found to have bilateral ptosis, ophthalmoplegia and midway pupillary dilation, otherwise the rest of neurological examination was within normal. Brain CT scan and MRI with contrast was performed which showed a sellar and suprasellar enhancing lesion replacing the pituitary gland with extension along the pituitary stalk and invasion of the cavernous sinuses measuring 4.3 × 2.3 × 1.8 cm which was suggestive of an invasive pituitary macroadenoma or a pituitary metastasis. (Figs. 1 and 2 ). He also underwent chest x-ray which has showed left lower zone opacification and whole-body FDG PET scan that has revealed a left parahilar mass in the left lower lung lobe most likely lung cancer associated with metastatic lymphadenopathy and likely metastatic left adrenal nodule and pituitary mass. (Fig. 3).

**Fig. 4:**
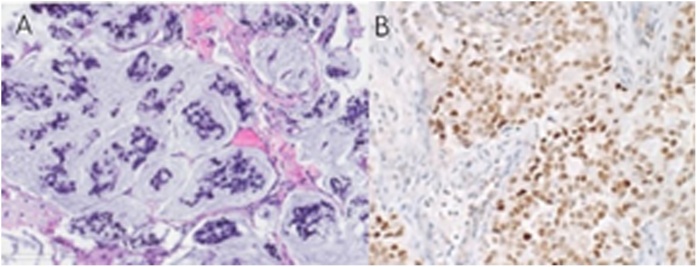
A) Pleomorphic epithelial cells in clusters floating in pools of mucin and separated by fibrovascular septae representing metastatic mucinous adenocarcinoma, H&E, 200×. B) Immunohistochemistry stain highlights the nuclear expression of TTF-1, which is in favor of metastatic lung adenocarcinoma, TTF-1 IHC, 20×.

With these clinical information, the patient underwent endoscopic trans-sphenoidal sellar mass surgery. Frozen section showed the mass to be a metastatic carcinoma and tumor debulking was done. The final histopathological report was metastatic non-small cell carcinoma/Adenocarcinoma with primary lung origin. It was positive for pancytokeratin, TTF-1, Napsin A, and CK7. After the diagnosis, patient was sent for definitive management. (Fig. 4)

## 3 Discussion

Metastatic disease to the pituitary gland is considered a rare condition. Although, studies of autopsies have found the percentage of pituitary metastases to be as high as 27 % when including microscopic metastases and metastases to the surroundings of pituitary gland, most patients die without diagnosis [2]. Pituitary metastasis account only for 1 % of surgeries to the pituitary gland [2,7]. The majority of case series that have been published about pituitary metastases showed that the two most common primary malignancies are breast and lung cancer. Other malignancies like colorectal, prostate, pancreas, and many others have been reported as well [3,8]. The reason behind the high percentage of breast and lung metastases to pituitary could be attributed to the fact that these are the two most common malignancies in female and male respectively and they are the most commonly reported tumors to metastasize to the brain [5]. In a case series with 52 patients diagnosed with pituitary metastasis published by Heshmati et al. the average age at time of diagnosis of a pituitary mass was found to be 60 years [3]. Around 84 % of pituitary metastases are in the posterior pituitary lobe; alone or in conjunction with the anterior lobe. It is thought that the reason behind the higher prevalence of posterior lobe metastasis is its direct blood supply in comparison with the anterior lobe which receives its blood supply indirectly through hypophyseal portal system [2,9].

In an autopsy study conducted by Silverman et al. only 7 % of pituitary metastases were symptomatic [10]. Different studies showed different percentages of the presenting symptoms however the most common symptoms include headache, ophthalmoplegia, visual field defect and pituitary dysfunction [2,3,11]. As the posterior lobe is more affected than the anterior lobe the most common hormonal abnormality is diabetes insipidus with an incidence of 61 %, other hormonal abnormalities are reported less frequently [2,7]. Pituitary metastasis can also be diagnosed incidentally in asymptomatic patients by radiological evaluation. In majority of cases patients are known to have malignant disease but in a small percentage such as our case the symptoms of the pituitary metastasis are what prompt the diagnosis of the primary malignancy and the metastasis. In a case series of pituitary metastases in japan, out of 157 cases 10.8 % detection of pituitary metastasis was initial to diagnosing the primary lesion [8].

The radiological modality of choice to evaluate a sellar mass is an MRI. Some of the features that suggest pituitary metastasis include being isotense on both T1 and T2, invasion of cavernous sinus, thickening of pituitary stalk, indentation at the diaphragma sellae giving a dumbbell shape, contrast enhancement and rapid growth in serial images. Most of these features are not specific making it difficult to differentiate pituitary metastasis from begin pituitary lesions [5,8,11,12].

Management of pituitary metastasis is complex and depends on many factors most importantly the extent and condition of the primary tumor. Endoscopic trans-sphenoidal excision and debulking procedures of pituitary metastases will not affect the survival but it will relieve improve symptoms namely headache and visual symptoms, quality of life and will provide a definitive diagnosis which is vital in cases where the clinical presentation is not suggestive of pituitary metastasis in order to prevent the unnecessary use of radiotherapy [7,11,13,14]. Another option for patient with limited survival is stereotactic radiation therapy [11]. Chemotherapy used specially in wide spread disease, however its effect on pituitary metastases have not been studied will [2]. The mean survival was found to be 17 months (0–240 months) in a case series study with 52 patients [3].

In most cases the diagnosis of pituitary metastasis is based on radiological findings in the presence of primary tumor and it is not confirmed by histopathology [5]. Moreover, with the advancement and use of PET/CT scan in staging of cancer more patients are diagnosed with sellar mass and because of the lack of definitive radiological distinctions between pituitary adenoma and pituitary metastasis more patients are subjected to empiric radiotherapy [5]. This approach is not accurate as seen in an autopsy study with 500 patients 18 were discovered to have pituitary metastasis and 9 were discovered to have pituitary adenoma [15].

## 4 Conclusion

Pituitary metastasis is a rare disease with no definitive features in clinical presentation and radiological images, making it difficult to diagnose. Its poor prognosis, especially when associated with wide spread malignancy, makes surgical resection and definitive diagnosis impractical in every case. However, endoscopic surgical resection and debulking procedure have an important role in alleviating symptoms, improving quality of life and confirming the diagnosis when clinical presentation does not strongly point to pituitary metastasis to avoid unnecessary radiotherapy and chemotherapy when possible.

## Declaration of Competing Interest

There is no conflict of interest regarding the publication of this paper.

## Sources of funding

There was no need of financial support.

## Ethical approval

Case reports does not require ethical approval in our institute.

## Consent

Written informed consent was obtained from the patient for publication of this case report and accompanying images. A copy of the written consent is available for review by the Editor-in-Chief of this journal on request

## Registration of research studies

Not applicable

## Guarantor

Dr. Ali Almomen, Dr. Aminah Alhashem

## Provenance and peer review

Not commissioned, externally peer-reviewed

## CRediT authorship contribution statement

**Aminah Alhashem:** Data curation, Writing - review & editing. **Mahmoud Taha:** Supervision. **Ali Almomen:** Conceptualization, Supervision, Writing - review & editing.
